# Attitudes and Knowledge of, and Preferences for Learning about Cultural Competence: A Study of Pharmacy Students from One Australian Pharmacy Program

**DOI:** 10.3390/pharmacy10030066

**Published:** 2022-06-20

**Authors:** Gloria Nkhoma, Chiao Xin Lim, Gerard A. Kennedy, Ieva Stupans

**Affiliations:** 1 School of Health and Biomedical Sciences, STEM College, Royal Melbourne Institute of Technology, Bundoora, Melbourne, VIC 3083, Australia; chiao.xin.lim@rmit.edu.au (C.X.L.); g.kennedy@federation.edu.au (G.A.K.); ieva.stupans@rmit.edu.au (I.S.); 2 Institute of Health and Wellbeing, Federation University, P.O. Box 663, Ballarat, VIC 3353, Australia; 3 Institute for Breathing and Sleep, Austin Health, 145 Studley Rd., Heidelberg, VIC 3084, Australia

**Keywords:** cultural competence, pharmacy curriculum, culturally and linguistically diverse populations, affective, cognitive, and behavioural learning, asylum seekers

## Abstract

Culturally and linguistically diverse populations, particularly asylum seekers, face challenges in accessing healthcare services. Pharmacists need to be capable of identifying and responding appropriately to the needs of diverse population groups. The aims of this study were to clarify student pharmacists’: knowledge of, and attitudes to, asylum seekers; their understanding of themselves with regard to cultural competence; their exposure to culturally and linguistically diverse clinical settings; their potential receptivity to learning opportunities directed towards cultural competence; and the extent to which they interpreted the current curriculum as improving their cultural competence. Pharmacy students’ viewpoints and perspectives were essential as emerging pharmacy professionals. This study employed mixed methods and convenience sampling. There were no significant (*p* > 0.05) associations between demographics and any of the survey items. Five themes emerged from the interviews: namely, exposure, formal vs. informal, positive views, conflict, and sufficiency. Pharmacy curriculum should ideally provide sufficient knowledge to meet culturally diverse healthcare consumers’ needs, especially asylum seekers. The most efficacious models for teaching cultural competence are as yet still undetermined. Interactive learning in cultural competence was recommended as essential.

## 1. Introduction

Healthcare systems heavily rely on pharmacist services, making the profession highly recognised and relied upon in developed countries [1]. The role of the pharmacist has evolved from a simple product dispensing practice to one that is patient-oriented and now includes medicine management, providing information on medicines, educating and counselling patients and providing basic medical tests and vaccinations [2]. In Australia, pharmacists must comply with professional standards, codes and guidelines [3]. Pharmacists possess unique and complex knowledge and expertise but also require excellent communication skills and cultural competence [3]. Pharmacists are expected to build partnerships with their consumers in order to establish trusting, respectful and culturally responsive working relationships for healthcare service delivery. For example, the Code of Ethics of pharmacy practitioners in Australia stipulates that pharmacists provide care to patients in a culturally safe and responsive manner [4].

Australia’s population has changed significantly especially over the past decade both culturally and linguistically [5], as evidenced by 2020 statistics [5]. Australia has a number of programmes that allow migrants to enter the country with a valid visa such as skilled migrant, family reunion, business and investment and humanitarian programs for refugee entry [6]. Asylum seekers may arrive in Australia with or without a valid visa. Those who arrive without visas have restrictions and may be placed in an immigration detention setting instead of mainstream community housing [6]. The diversity of the Australian population has a number of implications in terms of healthcare services provision. The challenges faced by culturally and linguistically diverse (CALD) populations, particularly asylum seekers, in accessing healthcare services are well documented in the literature [7,8,9].

Cultural competence has been reported as being critically important when dealing with CALD populations to bridge the health inequity gap [9,10]. Cultural competence is recognised as having five domains: (1) cultural awareness—recognition of cultural differences between one’s culture and that of others; (2) cultural knowledge—knowing cultures of other ethnic or cultural groups; (3) cultural skills—having skills that enable one to interact with people from diverse cultures; (4) cultural encounters—having been exposed to people of diverse cultural backgrounds; and (5) cultural desires—motivation of wanting to engage with people from diverse cultures to understand their culture [11,12]. These five cultural competence domains are linked to three broad domains of learning: affective, cognitive and behavioural. Affective learning—awareness and sensitivity to cultural values, needs and biases; behavioural learning—skills required to be effective in cross-cultural encounters; and cognitive learning—cultural knowledge and understanding of theory, research, and cross-cultural approaches to care [13].

For the attainment of cultural competence, the three key domains of affective, cognitive and behavioural learning need to be achieved at the individual level. Healthcare workers need to be cognisant of culture’s impact on health and consciously assess their own tendency of ethnocentricity, biases and prejudices. Integral to cognitive development is understanding the relationship between lived experience, stereotyping and empirical knowledge [14]. To improve cultural competence among healthcare workers, cognitive and affective domains are imperative as they have the potential to emphasise the student learning process and resistance or receptivity to cultural diversity concepts [15]. Multiple interpretations are associated with the domain of cultural awareness, ranging from a simple consciousness that differences exist and culture matters to a deeper commitment towards valuing diversity [16].

According to Wells Cultural Development Model [17], the affective phase implies the application of learned knowledge in the cognitive phase to achieve change in attitudes and behaviours. The preliminary step in moving from the cognitive to affective phase is a recognition of the ways in which our thoughts, perceptions, and impressions are shaped about people whose cultural backgrounds are different from our own [17]. Experiences with people from culturally diverse backgrounds and a willingness to embrace diversity rather than resist it, can serve as a way to facilitate progression through the cognitive and affective phases [17].

There is a need to embrace the cultural differences that may exist between healthcare providers and consumers that may conflict with the practitioner’s own views [18]. In order to effectively bridge potential differences in the process of attaining cultural competence, it is imperative for health professional students and health professionals to be able to identify and respond adequately to bias, and cultural miscommunications caused by differences in habits, values and communication styles among others [19]. A broad view and increased understanding of cultural diversity are essential from both the humanistic and pragmatic perspectives as they are both valid in the pursuit of increasing the understanding of cultural diversity [15]. Cognitive and affective levels may be achieved by a student through hearing a lecture on cultural diversity; however, this level does not assume that the student attains the skill but rather that the information has simply been received [15].

A review of media reports and government communications shows evidence of anti-asylum seeker sentiments and prejudicial attitudes towards asylum seekers in Australia, especially those arriving illegally by boat [20,21,22]. Evidence of widespread prejudice towards refugees and asylum seekers persists, even though the inclusion of a cross-cultural focus in university teaching has often been part of the requirements for professional accreditation, particularly in the health sciences [23]. Inappropriate views held by health professional students and health professionals towards refugees and asylum seekers could compromise the delivery of competent care to these groups [24].

In this study, the focus was on cultural competence, which is a broad concept that incorporates a number of domains: that is, cultural knowledge, awareness, skills, encounters and desires, which are all relevant for emerging healthcare professional students, to address the cultural needs of clients/patients from diverse backgrounds [9,10,11,12]. Cultural safety is creating a safe environment that addresses the inherent power imbalance between a healthcare provider and consumers, and cultural responsiveness focusses on healthcare services that respond to the cultural needs and backgrounds of consumers [25,26,27]. Cultural responsiveness and cultural safety both focus on how institutional care is both envisaged and delivered for Aboriginal and Indigenous people and Torres Islanders [25,26,27]. Based on what cultural responsiveness and safety entail, they are not synonymous with cultural competence but rather hallmarks of cultural competence, since they do not encompass all the five domains of cultural competence.

The aims of this study were to clarify: (1) the knowledge of, and attitudes to, asylum seekers by student pharmacists; (2) student pharmacists’ understanding of themselves with regard to cultural competence; (3) the exposure of student pharmacists to culturally diverse clinical settings; (4) student pharmacists’ potential receptivity to learning opportunities directed towards cultural competence; and (5) the extent to which students interpret the current curriculum as improving their cultural competence. In this study, the researchers focused on pharmacy students with the aim to capture viewpoints and perspectives from emerging pharmacy professionals.

## 2. Methods

This study employed mixed methods using both qualitative (semi-structured in-depth interviews) and quantitative methods (survey: convenience, targeted, cross-sectional). Participants were invited via emails sent by course co-ordinators to potential participants who were pharmacy students at Royal Melbourne Institute of Technology University in Melbourne, Australia (convenience sampling).

### 2.1. Quantitative Data Collection Process-Survey

For the survey, an electronic survey was disseminated via email, and the paper survey was distributed by the researcher in person to second- and third-year undergraduate pharmacy students together with the consent form. The survey was completed between October 2019 and May 2020.

### 2.2. Developing Survey Questions

Survey questions were developed by the current researchers from themes previously published in the literature and informed by the study aims and research questions. Researchers structured questions around previous research on host populations’ attitudes and perspectives on asylum seekers and their rights [22,28,29,30]. A process of evaluation was conducted by the researchers. Consensus was reached between researchers on the content of the final questions included in the survey to assure questions addressed the study aims. The survey included questions on demographics; age, sex and place of birth and parents’ country of birth (*n* = 3); on knowledge of asylum seeker health and entitlements (*n* = 7); and on views, attitudes and perspectives on asylum seeker health and social welfare entitlements (*n* = 10).

The 17 survey questions were categorised into knowledge as well as perspectives and views on the five domains of cultural competence: that is, cultural awareness, skills, knowledge, encounters and desires to assess the five domain(s) of cultural competence. Identifying existing knowledge through the survey established baseline knowledge, perspectives and views to inform potential intervention(s) types and to address knowledge gap(s) in the future. The rationale for assessing students’ knowledge on diseases prevalence and their views on healthcare entitlements was informed by the previously identified link between empathy, cultural awareness and competence [22,23] and the high disease burden of chronic-non-communicable diseases (CNCDs) such as diabetes and hypertension in this populace [9,10]. Empathy is known to foster cultural sensitivity, which is the building block for cultural competence [21,22,23]. Assessing the knowledge and understanding of the disease burden in this populace even though not comprehensive was identified as essential for raising awareness and empathy as a precursor for developing cultural competence [21]. The process of designing, structuring, validating and categorising the survey questions rather than using already validated survey questions was necessary due to the scarcity of tools specific to pre-registration pharmacy specialty as well as inherent restrictions and biases [19,31]. The process of categorising questions was discussed by all the researchers, and questions were categorised into cultural competence domains through assessing questions in relation to the definition of the domain. The main keywords in the question that aligned with a particular domain were identified and tagged to the question. Three researchers (GN, CL and GAK) conducted this process, and where consensus was not achieved, the independent researcher (IS) gave the final decision.

### 2.3. Qualitative Data Collection Process—Semi-Structured Interviews

Semi-structured in-depth interviews were conducted (GN) between August and November of 2020. The choice of using semi-structured in-depth interviews matched the purpose due to the potential to delve into sensitive and personal information [31]. Semi-structured interviews were used to allow in-depth exploration of the specific phenomenon, and the questions used were open-ended questions to allow the collection of rich data [32,33]. The interview guide consisted of open-ended questions with the option of refining the questions as interviews progressed in case of data saturation. Students were invited to contact the researcher by email or phone to arrange interviews. Interviews were conversational in nature, and there were ample opportunities for students to elaborate on answers to questions.

Interviews were audio-recorded and transcribed verbatim. The deductive thematic analysis approach where a pre-determined framework is utilised was used to analyse data [33]. The thematic framework was developed informed by Braun and Clarke [33] to make sure it conformed to reflexivity, rigour and expected quality [34,35]. The approach consisted of six steps: (1) familiarisation; (2) coding; (3) generating themes; (4) reviewing themes; (5) defining and naming themes; and (6) writing them up with supporting statements The six -stage process was initially undertaken by GN and repeated (CL and GAK). Emerging inconsistencies were addressed (IS). The familiarisation step entailed reading through the interviews to gain understanding, and the coding step involved highlighting text in every interview transcript that carried the main content of what was being said. The deductive method of generating themes was used, which involved grouping the codes depending on the theme and then generating the themes. In the next step, themes were reviewed per the topic of the interview and rephrased to fit the topic in question. Each theme was defined and named, conceptualising them in the context of the topic and relating them to the support statements from the interviews. All four researchers (GN, GAK, CL and IS) repeated the process of appropriately interpreting the matrix and facilitating the generation of descriptions, categories, explanations and typologies.

## 3. Results—Quantitative Data

### 3.1. Demographic Data

Demographic data collected through the survey included age, sex and place of birth/parents’ place of birth. The total of 134 students completed the survey, and the sample consisted of males (*n* = 34) and females (*n* = 100) aged 18–35. There were 128 (95.5%) students aged 18–25 years old, 5 (3.7%) students aged 26–33 years old and 1 (0.75%) participant aged 34+ years old. The majority of the 134 students were born overseas (but were not international students) (*n* = 63:47%), followed by students who were Australian born with one or both parents born overseas (*n* = 47:35%), and the least number (*n* = 24:18%) were Australian born with both parents born in Australia.

### 3.2. Summary of Results—Frequencies and Percentages of Students’ Responses to Questions

The majority of students correctly knew that: (1) Syria was the origin of most asylum seekers (86%), (2) asylum application status defined asylum seeker social welfare entitlements (98%) and (3) the difference between asylum seekers and refugees (95%). The facts on disease prevalence and how asylum seekers compared with host populations were correctly identified—60% correctly identified these as the most common diseases for asylum seekers.

The majority (80%) of the students thought asylum seekers arrive in host countries for a better life. However, the majority of students thought asylum policies were too hard (43%) and that asylum seekers should qualify to be in mainstream accommodation (67%). Most of the students ((70%) supported giving full healthcare access to asylum seekers and disease screening on entry for CNCDs, even though most of them (60%) had not engaged with asylum seekers. Most students (81%) acknowledged all healthcare workers had a duty to promote health and well-being in asylum seekers and identified themselves as highly likely to serve asylum seekers in the future (see Supplementary Table S1 for details of the questions, cultural competence domain(s) embedded within the questions, responses in frequencies and percentages).

### 3.3. Identifying Any Existing Correlations between Variables

Chi-square tests were conducted to examine whether any associations between variables such as age, sex or place of birth/parents’ place of birth and each response from the 17 questions were significant. None of the associations between the independent and dependent variables were significant (*p* > 0.05).

## 4. Qualitative Data

A total of eight pharmacy students were interviewed (four males and four females), and data saturation was attained upon interviewing the eighth participant. Immigration status included students from the three categories: Australian born, both parents born in Australia (*n* = 2), Australian born, one or both parents born overseas (*n* = 3) and born overseas, but not international students (*n* = 3). The five themes that emerged from the analysis were: exposure, formal vs. informal, positive views, conflict, and sufficiency. Interpretations of the students’ comments were based on how they related to the theme and relevance to the research aims.

- Theme 1: Exposure

The students attributed their cultural competence to associations either at workplaces, family or social contacts. They associated engaging with CALD populations as an essential element for developing the cultural competence requisite for pharmacy practice. The practical experience to develop cultural competence was linked to roles where students had worked in CALDs communities (*n* = 5). “*The cultural competence skills I have gained are mainly due to working five years as a pharmacy assistant.”* Where students had less exposure with people from CALD background, they perceived it as an inhibitor of becoming culturally competent. There was acceptance of low cultural competence due to the lack of exposure to CALD populations while working in some regional non-patient engagement services (*n* = 3). *“I’m cognisant of how much there is that I don’t know due to my limited experience with face-to-face care with patients where I’m working.”*

As such, most had a chance to encounter patients with different cultural backgrounds, which helped them understand cultural competence concepts and the capacity to identify areas where cultural competence was lacking.

- Theme 2: Formal vs. informal

All students (*n* = 8) demonstrated an understanding of what cultural competence entailed, since their definitions and explanations included some of the keywords used to describe cultural competence. However, all their definitions were based largely on self-learning (informal) and not from formal learning and were superficial in terms of understanding as pointed out; *“…the ability to be able to appreciate differences in terms of culture and values of people from different ethnicities and cultural backgrounds.”*

- Theme 3: Positive views

All participants identified positive outcomes from being culturally competent in practice as enhanced delivery and quality of medical care, better communication between patients and the pharmacist, increased medication compliance, patients becoming more comfortable with their culture even when receiving medical care and enabling the pharmacist to know what is and what is not allowed in a given culture. The following quote illustrates this view: “*Understanding their cultural barriers and their health literacy levels helps to get the maximum benefit of their medication.”*

Students (*n* = 4) identified that being sensitive about cultural issues—for example, taking medication during religious fasting—was beneficial and recommended the use of resources for guidance. As one student said, *“I would check with online resources.”*

- Theme 4: Conflict

Students (*n* = 8) were cognisant of the challenges they could face as pharmacists. A participant reiterated the importance of respecting patients’ culture which may differ from the practitioner’s by saying, “*I always respect their choice and uphold their cultural values.*”

However, students (*n* = 4) acknowledged the need to try and understand the patient without letting their beliefs interfere with their professional responsibilities. Moreover, some stated they had used their cultural competence skills on several occasions in offering services, and all thought it was quite useful for both the pharmacist and the patient, “*And by understanding your patient, then you can tailor your service and your counselling around them based on their cultural belief.”*

- Theme 5: Sufficiency

The limitations of traditional teaching methods were echoed by all students. Students (*n* = 8) stated that the current pharmacy curriculum tries to incorporate cultural competence in a very superficial way, for example, essay writing. They all agreed that there was not enough content to adequately address their cultural competence needs and identified it as inadequate.

*“I joined a leadership and cultural competence style workshop. I didn’t feel like I got anything out of them. I felt it was all a lot of just rhetoric and I kind of tuned out.”*

To address the insufficiency of the prevailing cultural competence curriculum, they all suggested a culturally inclusive education that entailed the incorporation of scenario-oriented teaching methodologies to reflect how to manage patients from different cultural backgrounds as one explained, *“… I think learning through a scenario-based learning approach is more beneficial.”* Students (*n* = 4) commented that there was a need to culturally diversify presenters who gave talks on cultural competence in healthcare settings.

*“I reckon it would be really helpful to have people from different cultural backgrounds giving talks and not only Indigenous people giving talks.”*

Students (*n* = 8) suggested having simulations, workshops tutorials, and talks as some of the ways cultural competence can be incorporated into the pharmacy curriculum. They, however, emphasised cultural competence as a comprehensive hands-on skill rather than just simply theories or explanations.

## 5. Discussion

### 5.1. Impact of Cultural Competence Domains Attainment Gaps

There was need to determine students’ knowledge and attitudes. Findings from this study have shown that the majority of students knew about asylum seekers’ place of origin, details of classification of asylum seekers and their disease prevalence. However, responses to some questions indicated a lack of political awareness.

It is well documented that there is a high disease burden of chronic non communicable diseases (CNCDs) such as diabetes, hypertension and respiratory diseases in asylum seekers [9,10,36,37]. CNCDs disease are often life-long diseases, and some require drastic changes in lifestyle [38], which may be a challenge for healthcare consumers, as they may try to balance their cultural beliefs with the expected healthy lifestyle. The challenges of improving treatment compliance and promoting a change of lifestyle such as diet are surmountable when healthcare professionals incorporate cultural competence with the knowledge on CNCDs prevalence to drive positive patient outcomes [9,10]. The above stipulated power of cultural competence and knowledge on CNCDs in this populace is vital for emerging pharmacists.

There were some negative views on why people seek asylum, appropriate asylum seekers’ placement upon arrival and their views on government policies for asylum seekers and on those who arrive by boat. This finding may be attributed to a number of reasons such as attainment of the cognitive domain of cultural competence without nurturing of the affective domain of cultural competence. The affective domain denotes a demonstration of aspects of cultural competence such as humility and empathy, while the cognitive domain entails understanding and knowledge [14]. Knowledge and understanding differ from empathy in that empathy can be simulated [37,39]. Although there were connotations of some negative views in students, our findings exposed no correlation between these views with gender, age, sex or place of birth/parents’ place of birth. The non-existence of the association between negative views and demographics creates the need to further investigate other potential associations of these views.

### 5.2. Implications of Cultural Competence and the Five Cultural Competence Domains on Clinical Practice

The healthcare needs of pluralistic and diverse populations have the potential to be met when professionals who provide the services are culturally competent. Cultural competence is known to bridge the gap of health inequalities between minority populations and the host population due to increasing healthcare services utilisation, community engagement and ultimately improving health outcomes [10,38]. The process of shifting the five cultural competence domains (cultural awareness, skills, knowledge, encounters and desires) through teaching and learning showed that it is not consistent for all domains [39]. This explains some of our findings, where the majority of students (>50%) showed some knowledge of cultural competence in the domains of knowledge and desires on questions focussing on ‘asylum seeker health entitlements and disease prevalence.’ The challenge of attaining efficacy in the cultural encounter domain is reiterated in the findings from a study by Musolino et al. [39], where the domain of encounters did not improve, and this was attributed to the domain not being able to be taught but rather being acquired, and it can only be shifted once there is an improvement in the other four domains: awareness, skills, knowledge and desires.

Some of the student responses to questions such as those based on asylum seekers’ living arrangements and on those who arrive by boat had some political connotations and some extent stereotypes and biases. These stereotypes and biases could be attributed to lower cultural competence skills and political views. It is documented that cultural competence skills and knowledge in healthcare professionals have the potential to eliminate stereotypes and personal biases [23]. All students who were interviewed in this study identified cultural competence as a driver for favourable patient health outcomes. Although they highlighted some challenges sometimes encountered in pharmacy practice due to differing cultural values and views, they still upheld the importance of tolerance, respect for other cultures and empathy, which forms part of their profession’s guiding principles. Equally important, exposure to culturally diverse communities was identified as crucial for harnessing strong cultural negotiating skills through interacting in an exponentially fast-growing and complex and diverse cultural environment.

### 5.3. What a Comprehensive Cultural Competence Curriculum Entails

Pharmacy students in this study raised concerns on the current curriculum where they said it was not appropriate in preparing them as culturally competent future healthcare professionals. Their main recommendations for the curricula to address their needs were: (1) a more hands-on learning approach, (2) the use of interactive methods such as simulations, vignettes, workshops, and (3) culturally diverse lived experience speakers among others as ways of teaching/learning cultural competence. Andrew et al. [40] assert that for healthcare students to become culturally competent, they should: master concepts within cultural competence (cultural skills and knowledge) and constructively critically self-reflect in affective domain areas, including feelings, values, attitudes, beliefs and motivations and on prejudice, bigotry, discrimination, and racism. As identified in a study on determining cultural sensitivities and attitudes towards refugees, there is a need for improving the intercultural sensitivity in healthcare students, especially attitudes towards refugees and asylum seekers [41]. There is a paucity of current research on cultural sensitivity, knowledge and attitudes of students and a clear understanding on the factors that underpin these relationships [41], which is an identified gap highlighted in our study.

Healthcare providers with exposure to culturally diverse and inclusive educational environments have the potential to have a strong inclination towards healthcare equity and positive culturally diverse interactions compared to those with minimum or no exposure [42]. It is worth noting that it can be challenging for healthcare professional educators to incorporate cultural dimensions in a highly diverse academic environment, as individual needs may differ depending on a number of factors such as previous knowledge and social contacts [43,44]. These previous findings coupled with our findings show that there is need for more research that focusses on designing curricula that can promote cultural competence in healthcare students, especially pharmacy students, and that promote positive health outcomes in CALD populations, especially asylum seekers and refugees. Results from some previous systematic reviews [45,46] are not compelling on which specific teaching/learning modalities can improve all domains of cultural competence. It is still undetermined which educational models are most effective and feasible in what specific contexts and groups and how many resources—for example, time—should be allocated for reaching the desired outcomes [47].

## 6. Limitations of the Study

This study was conducted at one university in Melbourne, Australia with students from one healthcare discipline (pharmacy). There is a potential for results to significantly differ if the study was to be conducted with students from a different discipline such as nursing or medicine or pharmacy students studying at a different university. The study utilised a survey and the topic guide, which were not validated tools with the potential of researcher bias. To mitigate this to some extent, all researchers conducted a thorough evaluation of the survey and topic guide questions. Some of the questions were informed by the existing literature on asylum seeker health and entitlements and cultural competence validated tools [28,29]. The purposive and convenient sampling technique utilised for the survey and interviews was ideal to ascertain that students fulfilled the research aims and findings, hence informing on the shaping of the future curriculum. Considering that students were from diverse cultural backgrounds and birth places, the information they provided was essential for informing on what is required for them to adequately serve CALD communities.

## 7. Conclusions

The students’ limited exposure to this population of interest was consistent to their responses where only 17% said they had previously engaged with this populace and identified the curriculum as not meeting their needs. There was an intriguing finding where there was non-existence of a relationship between age, sex or place of birth/parents’ place of birth and each response from the 17 questions. This highlights the need for further research on which factors influence which specific domains of cultural competence. Even though pharmacy students understood superficially what cultural competence entailed in theory, however, the practical aspect was lacking due to limited exposure to culturally diverse populations and a non-comprehensive cultural competence curriculum. Their cultural competence knowledge was mainly attributed to self-learning rather than formal learning through their current curriculum.

## 8. Implications on Current Practice

These results expose the knowledge gap created by the current pharmacy student curriculum being insufficient to provide students with requisite knowledge to meet the needs of culturally diverse healthcare consumers especially asylum seekers. The students’ recommendations on incorporating scenario-oriented methodologies, simulations, vignettes, workshops tutorials, and talks rather than the traditional lecture method require further researching in healthcare students especially pharmacy students due to these results not being too compelling.

## Data Availability

This study did not report any data.

## Supplementary Materials

The following supporting information can be downloaded at: https://www.mdpi.com/article/10.3390/pharmacy10030066/s1, Table S1: Responses on testing students’ knowledge on asylum seeker health entitlements and disease prevalence in asylum seekers: Responses on students’ views and perspectives on asylum seekers and their rights.
