# Supplementary material for: Attitudes and Knowledge of, and Preferences for Learning about Cultural Competence: A Study of Pharmacy Students from One Australian Pharmacy Program
Source: Pharmacy (Basel). 2022 Jun 20;10(3):66. doi: 10.3390/pharmacy10030066 (PMC9230435; doi:10.3390/pharmacy10030066)
Supplement: Supplementary file 1 [file pharmacy-10-00066-s001.zip › pharmacy-1733270-supplementary.pdf]

Supplementary Table S1: Responses on testing students' knowledge on asylum seeker health entitlements and disease prevalence in asylum seekers: Responses on students' views and perspectives on asylum seekers and their rights

Factual questions on asylum seekers	Cultural competence domain(s) embedded within the question	Responses *denotes the correct response	Frequency	Percentage
**Question (Q) 1.** Most asylum seekers in Australia come from which country?	*Knowledge and Desire*	**(a) Syria:*	86	64
		(b) Sudan:	33	25
		(c) Somalia:	15	11
**Q2.** What healthcare services are asylum seekers entitled to in Australia?	*Knowledge and Desire*	**(a) Entitlements differ depending on asylum application status:*	98	73
		(b) Free primary, secondary and tertiary healthcare services and free prescriptions:	23	17
		(c) None:	13	10
**Q3.** Which one is true about being an asylum seeker or refugee?	*Knowledge and Desire*	*(a) Asylum seekers have the right to reside in the country while refugees do not:*	22	16
		**(b) Refugees will have had their asylum application*	95	71

		accepted and given humanitarian protection:<br><br>*(c) The two terms mean the same thing:*	 <br>17	 <br>13
**Q4.** Please categorise non-communicable diseases in asylum seekers; Heart Diseases and Diabetes	*Knowledge and Desire*	*(a) Most common:*<br><br>**(b) Common:*<br><br>*(c) Least common:*	40<br><br>76<br><br>18	30<br><br>57<br><br>13
**Q5**. Please categorise non-communicable diseases in asylum seekers; Heart Diseases and Cancers	*Knowledge and Desire*	*(a) Most common:*<br><br>*(b) Common:*<br><br>**(c) Least common:*	23<br><br>70<br><br>41	17<br><br>52<br><br>31
**Q6**. Which chronic non-communicable disease in asylum seekers has a higher prevalence compared to the resident Australian population?	*Knowledge and Desire*	*(a) Asthma:*<br><br>**(b) Chronic pain:*<br><br>*(c) Diabetes:*<br><br>*(d) Heart disease:*	17<br><br>42<br><br>26<br><br>49	13<br><br>31<br><br>19<br><br>37
**Q7.** Please categorise non-communicable diseases in asylum seekers; Diabetes and Hypertension	*Knowledge and Desire*	**(a) Most common:*<br><br>*(b) Common:*	60<br><br>54	45<br><br>40

		(c) Least common:	20	15
**Questions on attitudes/perceptions towards asylum seekers and their social welfare entitlements**	**Cultural competence domain(s) embedded within the question**	**Responses**	**Frequency**	**Percentage**
**Q8.** *What do you think is the primary reason why people seek asylum?*	*Knowledge and Awareness*	*(a) Want a better life:*<br><br>*(b) Fear of persecution:*	107<br><br>27	80<br><br>20
**Q9.** *Do you think asylum seekers who come by boat should be allowed to claim asylum in Australia?*	*Desire*	*(a) Yes:*<br><br>*(b) No:*	111<br><br>23	83<br><br>17
**Q10.** *What do you think about government policies on asylum seekers?*	*Awareness*	*(a) Too hard:*<br><br>*(b) Too soft:*<br><br>*(c) I do not know:*<br><br>*(d) Just about right:*	57<br><br>5<br><br>55<br><br>17	43<br><br>4<br><br>41<br><br>13
**Q11**. *What do you think should happen when asylum seekers enter a country?*	*Awareness*	*(a) Placed in asylum seekers' accommodation:*<br><br>*(b) Placed in the community with everyone else:*	90<br><br>27<br><br>17	67<br><br>20<br><br>13

		(c) Placed in detention centres:		
**Q12.** *Which of the following do you think should be accessible to asylum seekers?*	*Awareness and Desire*	*(a) Medical support only:*<br><br>*(b) Medical, financial and educational support:*<br><br>*(c) None:*<br><br>*(d) Education only:*<br><br>*(e) Financial support only:*	24<br><br>103<br><br>2<br><br>4<br><br>1	18<br><br>77<br><br>1<br><br>3<br><br>1
**Q13.** *Which chronic non-communicable diseases do you think should be eligible for free healthcare entitlements including free prescriptions?*	*Awareness and Desire*	*(a) All of these:*<br><br>*(b) Cardiovascular diseases:*<br><br>*(c) Diabetes:*<br><br>*(d) Hypertension:*<br><br>*(e) Respiratory diseases:*	106<br><br>9<br><br>22<br><br>2<br><br>5	79<br><br>7<br><br>16<br><br>1<br><br>4
**Questions on addressing chronic non-communicable disease burden in asylum seekers**	**Cultural competence domain(s) embedded within the question**	**Responses**	**Frequency**	**Percentage**
**Q14.** *Do you think chronic non-communicable diseases should be screened for at entry?*	*Awareness and Desire*	*(a) Yes:*<br><br>*(b) No:*	99<br><br>35	74<br><br>26

**Q15.** *Whose responsibility do you think it is to provide self-care and preventative education on chronic non-communicable diseases to asylum seekers?*	*Awareness and Desire*	*(a) All of them:*<br><br>*(b) Charities:*<br><br>*(c) Nurses and GPs:*<br><br>*(d) Public Health Workers:*	108<br><br>4<br><br>13<br><br>9	81<br><br>3<br><br>10<br><br>7
**Questions on engagement with asylum seekers**	**Cultural competence domain(s) embedded within the question**	**Responses**	**Frequency**	**Percentage**
**Q16**. *Have you had contact previously with asylum seekers in work, social networks etc.?*	*Encounters and Desire*	*(a) Yes:*<br><br>*(b) No:*	54<br><br>80	40<br><br>60
**Q17.** *In your current or future work do you think there is a chance of having contact with asylum seekers?*	*Encounters and Desire*	*(a) Yes:*<br><br>*(b) No:*	117<br><br>17	87<br><br>13
